# The Possibilities Now before Us

**Published:** 1920-02-21

**Authors:** 


### GRADUATE EDUCATION.

### The Possibilities Now Before Us.

It is not the first time we have referred to a subject which has already received a very great amount of attention and which opens up possibilities only paralleled by the requirements of true preventive medicine which Sir George Newman and others have so clearly brought before the country. The steps already taken, the Fellowship of Medicine, etc., are all by now too well known and appreciated to call for more than a passing reference, but day by day come to hand, slowly it is true, tidings of preparations, steps in the right direction by the smaller institutions. It is with the object of drawing adequate attention to these valuable efforts and gaining for them the encouragement they deserve that we again open the subject.

In no profession is training of a post-graduate character of more importance than in that of medicine. The great majority of graduates pass into their active career, and with the exception of medical literature, gain no further instruction in modern medical science. A famous surgeon once remarked that it took at least ten years for any radical change in treatment to permeate the ranks of the entire profession. Such a state of affairs is deplorable, and no doubt the somewhat vexed question of compulsory post-graduate education will have to be settled in the near future. Many a busy practitioner would be only too glad to take the opportunity of such education, but at the present time it simply is not worth the trouble. Our great London hospitals are still chiefly used for undergraduate teaching, and such teaching must perforce be chiefly of an elementary character and not in keeping with the wants of the post-graduate and the further refinement of medicine.

### A Specified Post Graduate Hospital.

If post-graduate classes are started at the present hospitals they will only clash with the existing state of affairs. It is the teaching of the visiting staff which the post-graduate needs most, and their time is already severely encroached upon by their academic duties. If both under- and post-graduates attend the clinical instruction of the visiting staff, then the class becomes unwieldy and the best fruits of tuition are thus lost.

A post-graduate hospital would be the panacea for all these difficulties; such a hospital should be provided with a post-graduate resident staff, and a visiting staff of the greatest eminence could easily be provided from the wealth of talent which exists in and around the London area.

### Visitors from Overseas.

Before the war the cities of Berlin and Vienna were the "Meccas" of medicine; students travelled thither from all parts of the world in their search for knowledge. The fame of these Germanic cities was worldwide, and no medical man really interested in his work, could afford to miss the unrivalled post-graduate instruction of these centres. As a matter of fact this great game was chiefly brought about by propaganda, and the Teutonic instruction was composed in the main of a *réchauffé* of what the teachers themselves had gleaned in foreign countries; for original research of a successful character has never been the "forte" of the Teuton, rather has he by patient energy elaborated the genius of other nations.

### London as an International Centre of Medicine.

The Great War closed the schools and clinics of Germany and Austria, and since that time students from other lands have been thrown on each other's resources. In London, by the medium of the "Fellowship of Medicine," much valuable post-graduate instruction has been given to medical officers of the Dominion Forces and of the United States Army Medical Service. The resources of this great city have not been found wanting, and it has been proved to possess not only magnificent clinical resources, but also an ability to teach her post-graduates in a way which compares very well with the instruction supplied in the Germanic cities.

We repeat, the clinical material and the teaching ability are here, but we have no suitable treasure houses for our wealth: and until such are obtained we cannot expect London to vie with the magnificently equipped cities of Austria and Germany.

### Ample Room in London for a New Hospital.

That our great London hospitals are overworked and always full is common knowledge. A glance at the waiting-list of patients requiring admission show that in many cases these are too long. All this goes to show that a new post-graduate hospital would not injure the clinique of the existing hospitals in any way whatever, and to the general public such an institution would be a great boon.

There are large areas of greater London where such a hospital could be erected, without fear of encroaching upon the radius of supply of other institutions: such a district as that of Clapham and Balham would provide an ideal example.

### Finance.

In Germany and Austria the "Post-Graduate Scheme" is supported when and where necessary by a Government subsidy. In this country the great root principle of our hospital system is that they are voluntary, and supported by voluntary contributions.

At the present time we observe the wonderful way in which the voluntary system is repairing the war-stricken finances of our hospitals. In the same way we are sure that money could and will be raised to forward this ideal of post-graduate education. The public need instruction on this topic, and such letters as those which Professor Pollard and Messrs. Adami, Allbutt, Bertrand Dawson, Arbuthnot Lane, Makins, late Professor Osler, and Rolleston have sent to the daily Press will do much to secure this end.

